# Identification and Discrimination of Sulfated and Unsulfated Gut‐Hormone Biomarkers in Human Serum by Nanopore

**DOI:** 10.1002/smll.74968

**Published:** 2026-08-03

**Authors:** Luca Iesu, Laurent Bacri, Juan Pelta, Benjamin Cressiot

**Affiliations:** ^1^ CNRS, LAMBE, Université Paris‐Saclay, Univ. d'Évry CY Cergy Paris Université Cergy France; ^2^ CNRS, LAMBE, Université Paris‐Saclay, Univ. d'Évry CY Cergy Paris Université Évry‐Courcouronnes France

**Keywords:** biofluids, gastric cancer biomarkers, nanopores, peptides, PTMs

## Abstract

Cholecystokinin‐8, an octapeptide, is a gut hormone mainly involved in the digestive process and neuromodulation. Over the last four decades, its biological functions have been elucidated and shown to be critically modulated by tyrosine sulfation. Recently, this octapeptide has been identified as a potential biomarker in several tumours. Regrettably, there are no clinical or analytical methods capable of distinguishing between the unsulfated (CCK‐8) and sulfated (SO_3_‐CCK‐8) forms in controlled media (e.g., saline) or complex biofluids (e.g., human serum). Here, we use an aerolysin nanopore inserted in a lipid‐polymer membrane to discriminate them with high specificity and sensitivity at the single‐molecule level in both controlled media and complex biofluids, even at low nanomolar concentrations. We found that sulfated and unsulfated CCK‐8 generate highly resolutive electrical signatures. Analysis of these signals shows discrimination based on three independent parameters. Using a robust hybrid lipid‐coblock membrane, we successfully performed measurements in 20% (v/v) human serum, thereby enabling the identification of peptides differing by a subtle post‐translational modification. These results enable reliable discrimination of these biomarkers for diagnostic evaluation.

## Graphical abstract

This study discriminates between sulfated and unsulfated cholecystokinin‐8 gut‐hormone forms at single‐molecule‐level resolution. Using an aerolysin nanopore embedded in a hybrid lipid‐polymer membrane, the system produces distinct electrical signatures enabling highly sensitive detection, even in 20% human serum. This approach supports reliable biomarker discrimination for enhanced diagnostic evaluation of disease.

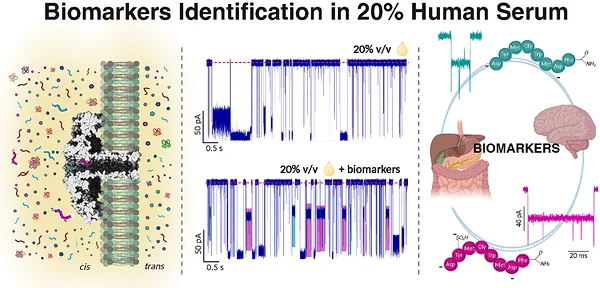

## 1 Introduction

In the coming decades, we can expect a higher incidence of disease [1]. Two primary reasons are the rise in pollution [2, 3] and the global increase in median age [4]. Early diagnosis is therefore crucial to enable timely and effective treatment [5, 6]. Variations in peptide biomarker concentrations are associated with numerous pathological conditions [7], and beyond changes in abundance, post‐translational modification (PTM) of the same peptide can elicit distinct physiological responses [8, 9]. However, current clinical methods lack the sensitivity and specificity required to detect and quantify such subtle variations. Furthermore, immunoenzymatic assays are generally unable to simultaneously detect a biomarker and distinguish between its post‐translationally modified forms. Recently, the nanopore approach with electrical detection has shown the ability to identify subtle protein and peptide conformation changes and PTMs [10]. In the present study, a small biomarker family is investigated by single‐molecule nanopore sensing.

Cholecystokinin‐8 (CCK‐8) is a gut‐brain peptide hormone with multiple physiological roles. It is fundamental to the digestive process and is synthesized and secreted by enteroendocrine I‐cells in the gastrointestinal tract and by neurons in the central nervous system (Figure 1a) [11]. Fatty acids and/or certain amino acids in the chyme are known to be the greatest stimulators of CCK‐8 release [12, 13] as well as the monitor peptide and the vagal acetylcholine signaling [14]. In response to a meal, it is rapidly released into the bloodstream, inducing gallbladder emptying, pancreatic enzyme secretion, gastric acid release, and intestinal motility [12]. In the central nervous system, CCK‐8 is the most abundant neuropeptide and acts as a neuromodulator and growth factor [15, 16, 17].

**FIGURE 1:**
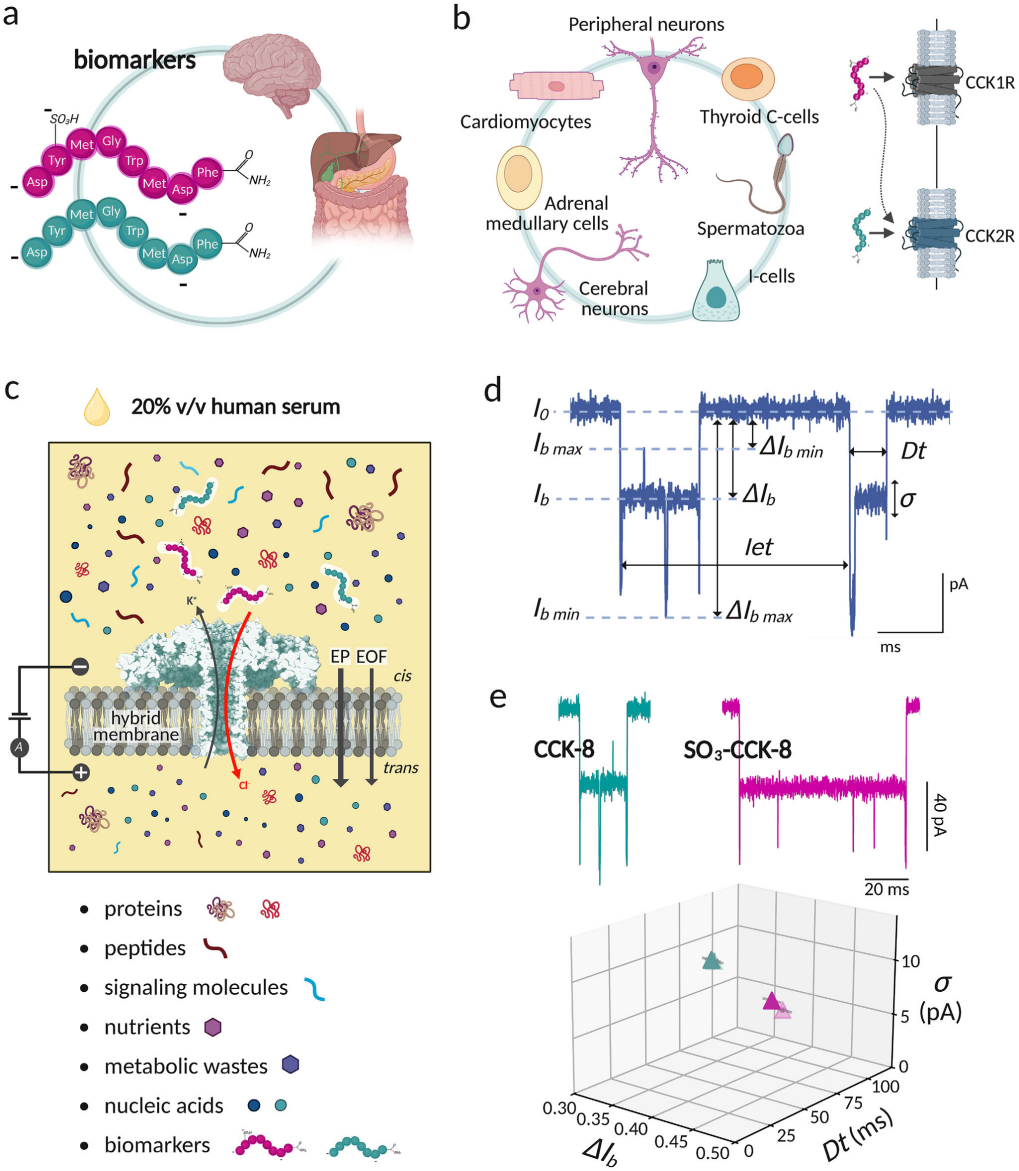
The diagnostic gut‐hormone biomarker CCK‐8: cell‐specificity and nanopore‐based three‐dimensional identification in human serum. (a) Schematic representation of unsulfated cholecystokinin‐8 (CCK‐8, teal; net charge ‐2e) and sulfated cholecystokinin‐8 (SO_3_‐CCK‐8, magenta; net charge ‐3e) together with their biosynthetic sites of production (duodenum, jejunum, and ileum in the digestive system; cerebral neurons in the brain). (b) Schematic representation of cell‐ and receptor‐ specificity of cholecystokinin‐8 biomarkers. (c) Schematic representation of the nanopore‐based direct identification system. Wild‐type aerolysin (PDB: 5JZT) is reconstituted into a hybrid lipid‐coblock (DPhPC‐PBD_11_PEO_8_) membrane separating two compartments filled with 20% v/v human serum in 3.2 m KCl, pH 7.5. Upon application of a +70 mV voltage bias, human serum components and the negatively charged biomarkers in the cis compartment are driven into the nanopore by the synergistic contribution of electrophoresis (EP) and electroosmotic flow (EOF). (d) Parameters extracted from a typical electrical recording: the open‐pore current (I_0_), blockade current (I_b_), average blockade level (∆I_b_= I_0_‐I_b_), average minimum blockade level (∆I_bmin_= I_0_‐I_bmax_), average maximum blockade level (∆I_bmax_ = I_0_‐I_bmin_), dwell time inside the pore (Dt), the inter‐event time (Iet), and standard deviation of the blockade signal (σ). (e) Examples of target biomarkers in 20% v/v human serum in 3.2 m KCl, pH 7.5. Bottom: three‐dimensional scatter plot of average blockade level (∆I_b_) versus average dwell time (Dt, ms) versus average standard deviation (σ, pA) with cones corresponding to CCK‐8 (teal) and SO_3_‐CCK‐8 (magenta). Each cone represents a data pool obtained from an equimolar mixture of both biomarkers at 250 or 62.5 nm. Mean values, standard deviation, and fitting parameters are reported in the Supporting Information. Images in panels (a), (b), and (c) were created using BioRender.

CCK‐8 receptors are widely distributed across tissues, including cardiomyocytes, adrenal medullary cells, I‐cells, thyroid C‐cells, spermatozoa, and both central and peripheral neurons (Figure 1b) [18, 19]. Importantly, receptor affinity is critically modulated by tyrosine sulfation. The type 1 cholecystokinin receptor (CCK1R) selectively binds sulfated CCK‐8 (SO_3_‐CCK‐8) with high affinity (Figure 1b) [20, 21], whereas the type 2 cholecystokinin receptor (CCK2R) binds both SO_3_‐CCK‐8 and unsulfated CCK‐8 (Figure 1b) as well as Gastrin17 [20, 21, 22]. The response evoked by the CCK1R, primarily expressed in the gastrointestinal tract [18], consists of the above‐mentioned effects that are part of the digestive process. On the other hand, CCK2R, which is mainly found in the brain and stomach [18], is generally involved in digestion (gastrin‐ergic acid secretion) [23]. CCK2R is also expressed on pancreatic islet cells, where it contributes to the secretion of insulin and glucagon (the incretin effect) [24].

Recent studies have shown that circulating levels are significantly reduced in both the early and progressive stages of gastric cancer, with approximately 146 ± 3 pg/mL in healthy controls versus 103 ± 2 pg/mL in patients [25]. Notably, they showed that levels do not significantly differ across cancer stages, suggesting their potential utility as an early biomarker [25, 26]. Beyond its potential application as a gastric cancer biomarker, the octapeptide is part of a complex signaling network [27] which must be explored in the next decades [28], highlighting the urgent need to develop more sensitive assays. Recently, CCK‐8 has also been proposed as a key biomarker for assessing malnutrition risk in older adults [29].

Another noticeable point is the autocrine effects of these peptides. C‐cells synthesize a large amount of CCK‐8, which binds to CCK2R expressed on the same C‐cell membrane [30, 31, 32]. Furthermore, the CCK‐8 peptide is expressed in various tumors (gastroenteropancreatic [33, 34, 35], medullary thyroid C‐cell [31], pituitary [36], pediatric [37, 38], astrocytomas [39], and gliomas [40, 41]) as well as in cell lines from small‐cell lung cancers [42, 43, 44]. As reported in recent work [27], autocrine effects of cholecystokinin peptides may significantly contribute to malignant tumor cell growth. Despite its clinical relevance, the reliable detection and discrimination of sulfated and unsulfated CCK‐8 remain unresolved. These challenges underscore the need for a method that ensures high precision and reliability in detection, discrimination, and quantification, such as the nanopore‐based approach [45].

Current detection methods are immunoassays, which lack sensitivity within the physiological range (pM) [9] and exhibit cross‐reactivity with gastrin [46], which is present at ∼5–10 × higher concentrations in human samples [47,48], a parameter that cannot be ignored when complex biofluids represent the starting specimen. Moreover, most enzyme‐linked immunosorbent assay (ELISA) do not distinguish between sulfated and unsulfated forms. Effectively, an ELISA CCK‐8 test [49] has a limit of detection equal to 12.35 pg/mL (≈ 10.8 pM). Although Liquid Chromatography coupled to Mass Spectrometry (LC‐MS/MS) can accurately differentiate peptide isoforms [50], the lability of the sulfate group during ionization hampers accurate quantification [47]. Additionally, to facilitate detection of small concentration changes, samples are pre‐treated by chromatography [51]. Furthermore, even if the biomarker under investigation can be quantified using an isotope‐based or unlabeled approach, MS is known to require at least 10^6^ copies of the targeted biomarker [52, 53], and results are obtained from averaged bulk analyses, which could potentially mislead a comprehensive understanding of healthy patients. Therefore, the discrimination between the sulfated and unsulfated forms in clinical samples remains unresolved, as do their exact concentrations. Under these circumstances, detecting and discriminating between these two biomarkers in biofluids is particularly challenging and technically demanding. Single‐molecule techniques can collect information every time the analyte generates a signal. Given the possibility to perform direct analysis without pre‐treatment, even for complex biofluids, the aforementioned constraints position the nanopore‐based approach as an eligible candidate to address these challenges [10]. Over the past three decades, nanopore technology has undergone a huge expansion, emerging as a highly versatile single‐molecule platform [45, 54, 55, 56]. Briefly, a single channel (nanometer scale) is reconstituted into a membrane that separates two chambers containing electrolyte (Figure 1c). Upon application of a voltage bias, electrolyte ions traverse the pore, resulting in an open‐pore current (*I_0_*; Figure 1d). When an analyte obstructs ion transport through the pore, a drop in the ionic current—known as a blockade event—is observed (Figure 1d,e). The blockade amplitude (*∆I_b_*) is mainly dependent on the analyte size relative to the pore diameter. On the other hand, the analyte's charge, along with the magnitude of the applied voltage bias, determines the duration of the blockade event (*Dt*) [57].

Nanopore approaches have been employed to probe peptide and protein sequences and conformations [58, 59, 60], as well as to investigate proteinaceous biomarkers [61, 62]. The aerolysin from *Aeromonas hydrophyla* is suitable for the investigation of small analytes, such as amyloid *β*‐peptides [63, 64], ginsenosides [65], and, more recently, the neurohormone oxytocin and its structural variants [66]. In this work, we use the wild‐type aerolysin as a single‐molecule nanopore sensor to detect and discriminate the CCK‐8 biomarkers with high specificity in a complex sample (Figure 1c–e).

Nanopore‐based single‐molecule approaches can be used to detect target analytes in complex samples using various strategies. In indirect methods that incorporate a purification step, nanopore measurements are preceded by the selective isolation of the target analyte from serum components by protein‐protein or protein‐ligand interactions (e.g., antibodies or aptamers) [67].

This approach enabled the detection of multiple serum biomarkers, including thyroid‐stimulating hormone [68], prostate‐specific antigen (PSA) [69], carcinogenic embryonic antigen [70], and HIV p24 [70].

By avoiding purification, other nanopore strategies still use binding partners to sense SARS‐CoV‐2 spike protein indirectly [71], PSA [72], epidermal growth factor [73], C‐reactive protein [74], thrombin [75], glucose and asparagine [76], and thiamine [77]. Relevant enzyme activity, such as alkaline phosphatase, has also been detected [78].

As previously reported, biofluids can interfere with lipid bilayers, thereby precluding nanopore measurements at reasonable dilutions [71, 77, 78, 79, 80, 81, 82, 83, 84, 85]. The direct detection of analytes in biofluids (blood or its derivatives) remains a significant challenge, given the limited number of examples in the literature to date. These include methylcytosine detection along DNA sequences [79], natriuretic peptides [80], and mutant hemoglobin [83].

Human serum is a complex biofluid composed of numerous compounds, including peptides, proteins, nutrients, nucleic acids, and small molecules [86], which disrupt bilayer stability and, in most cases, induce its rupture. The most abundant serum protein, albumin (about 40 g/L) [87], is known to absorb onto surfaces, whereas lipids (e.g., cholesterol esters, free fatty acids, phospholipids) [86] can interact with the lipidic component in the hybrid membrane. In addition, enzymes such as phospholipase can hydrolyze glycerophospholipids, which in the hybrid membrane represent the lipidic counterpart. These continuous interactions are indeed responsible for the membrane rupture. Therefore, owing to the well‐known challenges of measuring blood‐derived analytes using nanopores, direct discrimination of relevant peptides has not yet been explored at concentrations above 2% v/v in human serum [85]. Interestingly, defibrinated sheep blood measurements have been performed at 25% v/v concentration. However, the pore is characterized by an entropic gate and linkers that enable recognition of the specific target protein (streptavidin), thereby relying on selective binding [88].

## 2 Results and Discussion

Given the recent advancements in hybrid membrane stability [89], electrical measurements with wild‐type aerolysin were performed using a hybrid membrane obtained by mixing the (DPhPC) with the diblock copolymer poly(butadiene)‐b‐ethyleneoxide (PBD_11_PEO_8_) (1:1 w/w, 10 mg/mL in decane) (Figure 1c,e). The use of this kind of membrane enabled the direct detection of proteins in blood due to its greater stability compared to classical lipid membranes [88]. Employing this strategy, we achieved three‐dimensional separation of the sulfated and unsulfated forms of CCK‐8 in 20% v/v human serum with wild‐type aerolysin (Figure 1e). We characterized the two biomarkers in a saline solution (4 m KCl, 25 mm Tris HCl, pH 7.5 or 3.2 m KCl, 25 mm Tris HCl, pH 7.5 to match the dilution after addition of serum in the next part of the manuscript) under different voltage conditions (Figures S1–S7). At +40 mV, CCK‐8 and SO_3_‐CCK‐8 exhibit identical blockade levels and nearly the same dwell times (Figure S1). As the applied voltage increases, the two parameters differ between the two peptides (Figure S1). An additional population is observed for both peptides at +40 mV (Figures S2 and S3), but it does not enable discrimination. Analysis across all voltages enabled understanding of the dynamics of peptides with different net charges (‐2*e* for CCK‐8 and ‐3*e* for SO3‐CCK‐8, where *e* is the elementary charge) in aerolysin. Up to +80 mV, the dwell times are similar. However, above +80 mV, the voltage is high enough to transport SO_3_‐CCK‐8 charged at ‐3*e*, but not CCK‐8 charged at ‐2*e*. This difference in behavior becomes more pronounced as the voltage increases. For example, at +120 mV, the dwell times are 67 ± 7 and 15 ± 0.5 ms for CCK‐8 and SO_3_‐CCK‐8, respectively (Figures S4 and S5). Indeed, SO_3_‐CCK‐8, which has an additional negative charge, exhibits a shorter dwell time above +80 mV (Figure S1). This means that both peptides are in a trapping regime below +80 mV, whereas only SO_3_‐CCK‐8 begins to translocate as the voltage bias is increased further (Figure S1), consistent with its higher net negative charge. Additionally, in a recent study by Long's group, sulfation of tyrosine appears to alter the peptide's behavior within the pore and its relative signal [90]. While enhancing the blockade amplitude, sulfation likely directly influences peptide‐pore interactions, thereby resulting in different residence times.

After validating discrimination between equimolar and non‐equimolar mixtures at multiple voltages (Figures S6 and S7), we tested the challenge of the direct CCK‐8 biomarker discrimination in 20% v/v human serum.

The approach was benchmarked by adding a known volume of non‐diluted, non‐filtrated human serum to the saline solution to achieve a final dilution of 20% v/v (Figure 2a). Serum was added to both compartments to maintain osmotic balance and prevent membrane rupture. Due to dilution of the starting solution, the open‐pore current decreases (Figure 2b). Human serum exhibits multiple distinct blockade events, as indicated by the current trace and the scatter plot (Figure 2b,c) when measured with aerolysin. In serum, numerous components are present, most of which are highly negatively charged. Often, the current drops till the baseline at zero (Figure 2b, Figure S8). This means that a clog of charged components completely blocks the pore (Figure S8). To unblock the pore, we must invert the applied voltage from positive to negative and then back to positive. Due to the current drops relative to the baseline and the voltage switching, the traces require a specific cleaning process before analysis (Supporting Information, Methods part; Figures S9 and S10) to extract the different parameters (Figure 1d).

**FIGURE 2:**
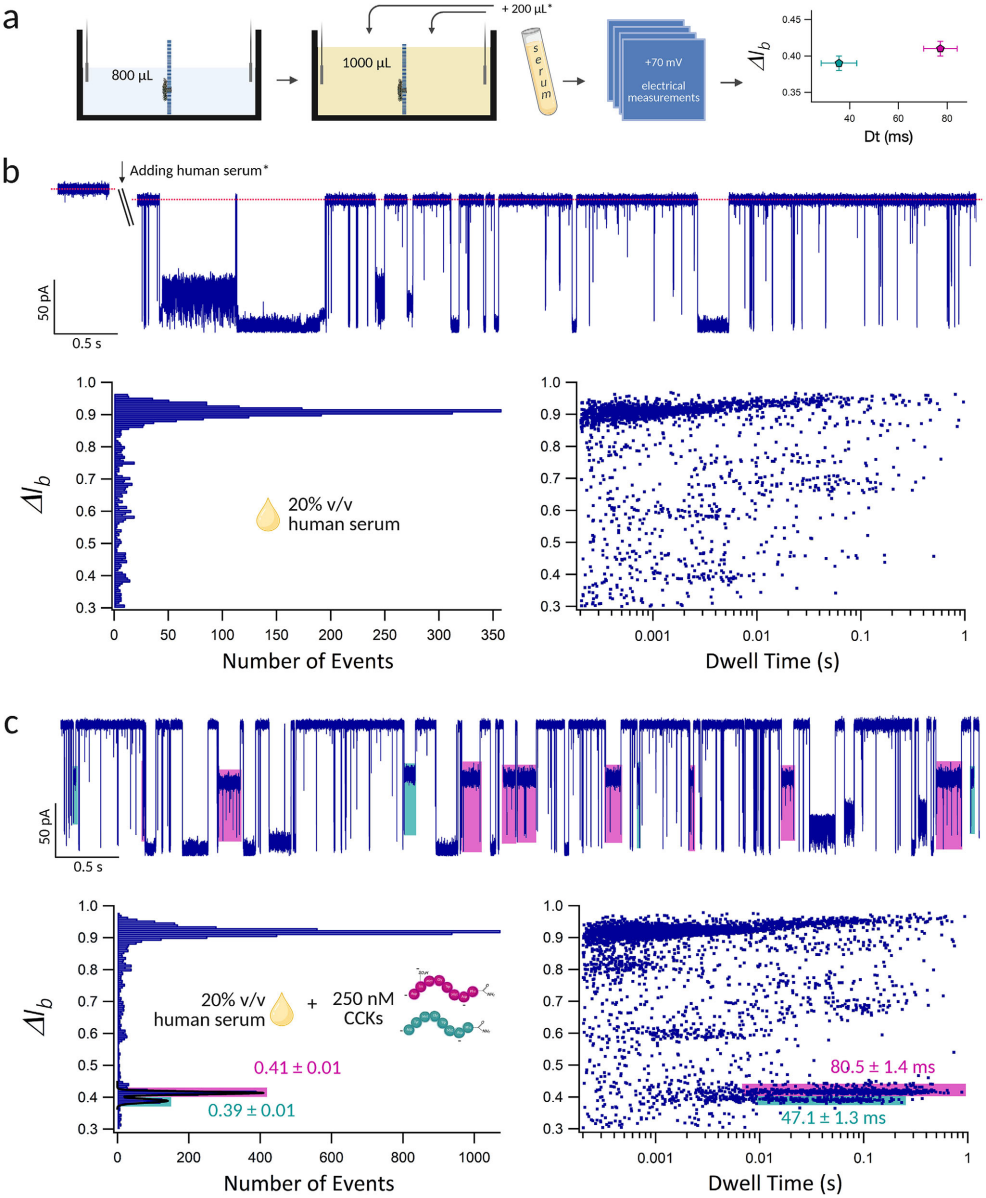
Identification of unsulfated CCK‐8 and its post‐translationally sulfated form SO_3_‐CCK‐8 in 20% v/v human serum using an aerolysin nanopore. (a) Schematic representation of the nanopore‐based approach used to discriminate CCK‐8 and SO_3_‐CCK‐8. After aerolysin insertion, 200 µL of human serum is added to both compartments, each containing 800 µL of 4 m KCl and 25 mm Tris‐HCl (pH 7.5). Electrical measurements are performed, followed by signal analysis, which enables discrimination of biomarkers: CCK‐8, teal; SO_3_‐CCK‐8, magenta. (b) Top: representative current trace of wild‐type aerolysin open‐pore current illustrating its lowering after adding human serum to the system, and its consecutive events. Bottom: left, histogram of the normalized average blockade level (∆I_b_) versus the number of events; right, scatter plot of normalized average blockade level (∆I_b_) versus the dwell time (Dt). Conditions: 20% v/v human serum in 3.2 m KCl, pH 7.5, under a +70 mV applied voltage. (c) Top: representative current trace of wild‐type aerolysin illustrating distinct blockade events associated with CCK‐8 (teal) and SO_3_‐CCK‐8 (magenta) within the human serum background. Bottom: left, histogram of the normalized average blockade level (∆I_b_) versus the number of events, fitted with a bi‐Gaussian (black line) function. Bottom: right, scatter plot of normalized average blockade level (∆I_b_) versus the dwell time (Dt). Populations corresponding to CCK‐8 (teal) and SO_3_‐CCK‐8 (magenta) are highlighted. The bi‐Gaussian fit confirms the most probable average blockade levels for each biomarker population (shown in the figure). Dt fitting parameters are reported in the Supporting Information. Condition: 250 nm equimolar mixture of CCK‐8 and SO_3_‐CCK‐8 in 20% v/v human serum in 3.2 m KCl, pH 7.5, under a +70 mV applied voltage. Panel (a) and the infographics in (b) and (c) were created using BioRender.

To avoid potential bias, we measured human serum without the target biomarkers (Figure 2b). Then, we measured it by spiking a known concentration of both biomarkers (Figure 2c). Interestingly, the aerolysin wild‐type has a satisfyingly high resolution to discriminate and identify the peptides here investigated directly; the background (Figure 2b) does not represent an obstacle to their discrimination and identification (Figure 2c). Specifically, the two biomarkers are discriminated bidimensionally by the values of *∆I_b_* and *Dt*, as shown in the histogram and scatter plot (Figure 2c). As shown previously (Figure 1e), to enhance discrimination robustness, we used a third parameter, the standard deviation (𝜎) (Figure 1d, Figures S11 and S17). As noted above, the two biomarkers exhibit similar blockade depths; however, in 20% human serum, the distinct *𝜎* values (Figure 1e, Figures S11 and S17) provide an additional discriminative parameter frequently used in machine‐learning classification [91]. Accordingly, 𝜎 may serve as an orthogonal feature relative to *∆I_b_* and *Dt*, particularly when these parameters converge or broaden due to the complex serum background, thereby limiting reliable discrimination. Consequently, we studied the effect of 20% v/v human serum on the blockade signals and their frequency (Figure 3).

**FIGURE 3:**
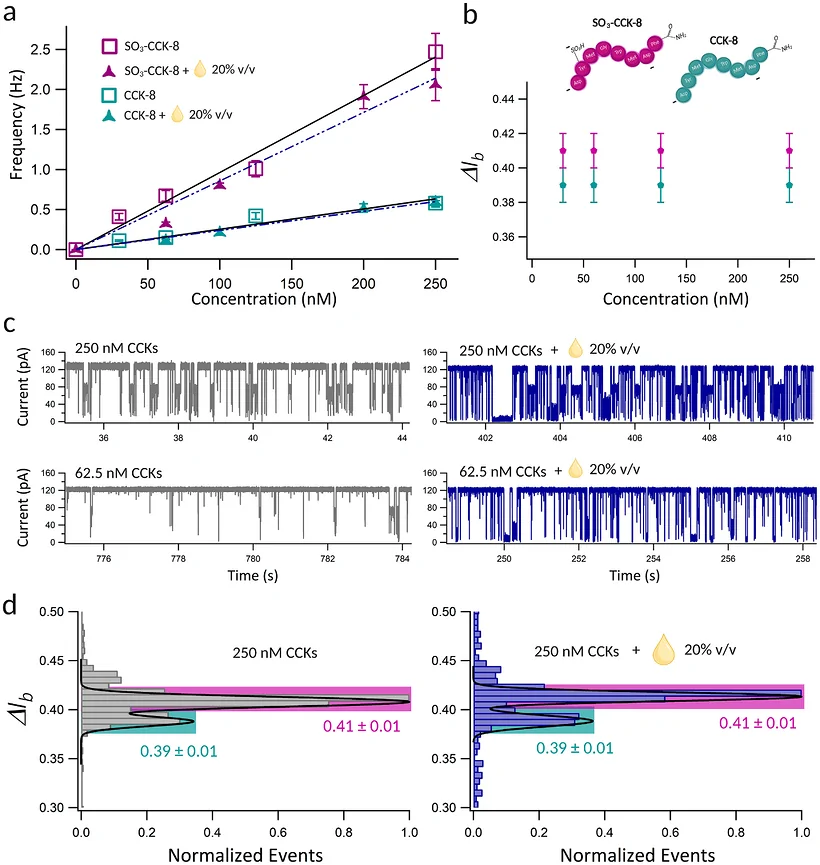
Effect of 20% v/v human serum on CCK‐8s blockade signals and event frequency. (a) Event frequency of CCK‐8 (teal) and SO_3_‐CCK‐8 (magenta), as a function of their concentration, under a fixed +70 mV applied voltage in 3.2 m KCl, 25 mm Tris HCl, pH 7.5, or in 20% v/v human serum in 3.2 m KCl, pH 7.5; linear fits are shown as black solid lines (buffer condition) and black dotted lines (human serum condition), respectively. Slopes for buffer condition are 9.62 ± 0.43× 10^−3^, and 2.53 ± 0.21× 10^−3^ nm ^−1^.s^−1^ for SO_3_‐CCK‐8, and CCK‐8, respectively. Slopes for human serum conditions are 8.56 ± 0.45× 10^−3^, and 2.4 ± 0.1× 10^−3^ nm ^−1^.s^−1^ for SO_3_‐CCK‐8, and CCK‐8, respectively. (b) Bidimensional plot of the normalized average blockade level (∆I_b_) for CCK‐8 (teal, 0.39 ± 0.01) and SO_3_‐CCK‐8 (magenta, 0.41 ± 0.01) calculated under different concentrations. (c) 10‐s snapshots of typical current traces showing distinct blockade events under different conditions: left, buffer condition; right, human serum condition. (d) Histograms of the normalized average blockade level (∆I_b_) versus the normalized number of events; left, buffer condition; right, human serum condition. Infographics were created using BioRender.

To corroborate our discrimination in human serum, we performed additional measurements at the same salt concentration (3.2 m KCl; Figures S12, S13, S15, S16) when human serum was added to both chambers. Blockade frequencies increase linearly with peptide concentration, consistent with Fick's law in the presence or absence of serum. The slopes are similar and are slightly modified by the addition of serum (Figure 3a). Similarly, blockade levels are consistent with concentration (Figure 3b; Figures S14–S16) and are unaffected by the presence or absence of human serum (Figure 3d; Figures S14–S16). Even if the frequency of SO_3_‐CCK‐8 differs slightly between the two conditions, this is not problematic, as the linear dependence on concentration is maintained (Figure 3a). These results indicate that human serum components do not interfere with the target peptides in a direct discrimination approach. These results notably suggest its applicability to clinical studies, given the absence of a reliable method for distinguishing between these two biomarkers.

Our group has already demonstrated the feasibility of detecting and discriminating among biomarkers in human serum at a 1:50 dilution [85]. Herein, we report the direct identification in such a low dilution of human serum (1:5) of relevant peptide biomarkers at physiological conditions by the nanopore approach, which has not been demonstrated yet. This is remarkable, given the urgent need to develop diagnostic methods [45, 92] and to determine CCK‐8 concentrations in clinical samples. Concomitantly, we rely on unmodified wild‐type nanopores rather than complex engineered nanopores. Engineering complex, analyte‐specific nanopores, as well as using adapters or ligand partners, limits the potential for multiple biomarker detection. This could represent a limitation for future integration of the nanopore into point‐of‐care (POC) tests [45].

## 3 Conclusion

We demonstrated that we can directly discriminate between the sulfated and unsulfated forms of CCK‐8 across a low‐concentration range, not only in a saline solution but also in complex biofluids. Although the measurements reported here were conducted at high ionic strength to achieve a higher signal‐to‐noise ratio, future studies should evaluate the impact of electrolyte composition at relevant biofluid concentrations. A deep understanding of these variations will be essential for assessing optimal performance, with the aim of facilitating its translation into clinical application. These results provide a starting point for discriminating between these two biomarkers in clinical samples, enabling their reliable quantification. However, as reported elsewhere [88, 89], hybrid membranes are not stable for more than ∼10 min, which represents a potential barrier to integration in microfluidic systems for POC applications, while still leaving the option of their use in clinical assays. Alternative polymers, such as poly(2‐methyloxazoline) (PMOXA) or poly(dimethylsiloxane) (PDMS) [93], or solvents, should be explored to improve the stability of the hybrid membrane. In this regard, a smaller aperture diameter (<150 µm) can drastically reduce the bilayer's surface area, thereby reducing its susceptibility to mechanical stress. Furthermore, the permanent Parylene C coating of the support can avoid the time‐consuming precoating steps required for Teflon‐ or other fluoropolymer‐based supports [94] and may lead to more stable hybrid bilayers. Thus, the next challenge is to develop a system that enables measurement of biofluids while providing robust support for the nanopore, thereby preventing components that destabilize it. Interestingly, such a strategy has already been developed, consisting of an engineered viral portal protein integrated into a solid‐state support for detecting proteins, peptides, and nucleic acids [95]. However, this system has not been tested for complex biofluids.

In this framework, the adoption of a multiplexed aerolysin‐on‐a‐chip system could enable detection at sub‐nanomolar concentrations. A recent proof‐of‐concept from Ying's group demonstrated improved detection limits using a multichannel aerolysin‐on‐a‐chip system based on the droplet interface method [96]. Notably, the same group also developed a nanopore multiplex sensor that integrates a series of orthogonal nanopores, enabling multiplex biomarker detection [97]. For a potential POC integration, further strategies must be explored to reach the femto‐ or picomolar range. One promising approach is the use of asymmetric salt conditions, for example, introducing Mg^2+^ on the trans side, which increased the capture frequency by 16.5‐fold compared to symmetric conditions [98].

## 4 Experimental Section/Methods

### 4.1 Production and activation of Aerolysin WT

Wild‐type proaerolysin was produced as described in previous works. In brief, after periplasmic expression of a C‐terminally His‐tagged protein in *E. coli* BL21‐Rosetta2 (MilliporeSigma, Burlington, MA, USA), cells were disrupted by osmotic shock to harvest the protein. Then, two steps of purification, nickel affinity and buffer exchange chromatography (Cytiva, Marlborough, MA, USA), were executed to obtain a final concentration of ∼1.7 *µ*M proaerolysin stored in 350 mM NaCl at 4°C until next use. Before use, proaerolysin monomers were activated using trypsin immobilized on agarose beads (Thermo Scientific, Waltham, MA, USA) for 15 min at RT.

### 4.2 Nanopore measurements

Resistive pulse experiments were performed by using a vertical planar lipid bilayer setup (Warner Instruments, Hamden, CT, USA). The electrolyte solutions were different in ionic strength: 25 mM Tris, pH 7.5, in 4 M or 3.2 M KCl.

For experiments in controlled media, a 1% (w/v) stock solution of 1,2‐diphytanoyl‐sn‐glycero‐3‐phosphocholine (DPhPC; Avanti Polar Lipids LLC, USA) in *n*‐decane (Sigma Aldrich, St. Louis, MO, USA) was used to paint in a 150 µm aperture a lipid membrane separating two compartments filled with 1 mL of electrolyte solution. Once a single aerolysin nanopore was inserted into the *cis* compartment, CCK‐8 and SO_3_‐CCK‐8 (AnaSpec, Fremont, CA, USA) were added to the same compartment and gently mixed at the appropriate concentration for the experiment.

For human serum measurements, a 1% (w/v) stock solution in *n*‐decane of a 1:1 mixture of DPhPC and poly(1,2‐butadiene)‐b‐poly(ethylene oxide) (Polymer Source Inc, QC, Canada) ‐indicated as PBD_11_PEO_8_ in the main text—was used to paint in a 150 µm aperture a hybrid lipid‐coblock membrane separating two compartments filled with 800 µL of electrolyte solution. Once a single aerolysin nanopore was inserted into the *cis* compartment, 200 µL of pooled human AB serum (MP Biomedicals, Irvine, CA, USA), either biomarker‐spiked or non‐spiked, was added to both compartments and gently mixed. Serum was added to both the *cis* and *trans* compartments to prevent osmotic imbalance.

### 4.3 Data acquisition

A potential difference was applied across Ag/AgCl electrodes (Sigma‐Aldrich, USA) to measure the current signal using an Axopatch 200B amplifier (Molecular Devices, LLC, San Jose, CA, USA). Data were recorded at 250 kHz intervals (4 µs sampling time) and filtered at 5 kHz using a DigiData 1440A digitizer (Molecular Devices, LLC, San Jose, CA, USA) and Clampex software (Axon Instruments, Union City, CA, USA).

### 4.4 Data analysis

Igor Pro Software and in‐house algorithms were used to process and analyze data, and to generate plots (Wavemetrics Inc., Portland, OR, USA). The standard deviation of the electric signal (*σ*) was used in the calculation *I_0_* − 3*σ* for defining the mean open‐pore current. A second calculation *I_0_* − 5*σ* was applied to define the blockade event. Each blockade event was characterized by three parameters (Figure 1d): the open‐pore current (*I_0_*), the average blockade current level (*I_b_*) within the event, and the event duration (*Dt*). The inter‐event time (*Iet*) is the time between the onset of two distinct blockade events. The *I_b_* was used to calculate the mean blockade level *∆I_b_* = (*I_0_* – *I_b_*)/*I_b_*; the *I_bmax_* was used to calculate the mean minimum blockade level *∆I_bmin_* = (*I_0_* – *I_bmax_*)/*I_b_*; the *I_bmin_* was used to calculate the mean maximum blockade level *∆I_bmax_* = (*I_0_* – *I_bmin_*)/*I_b_*. Every blockade level was normalized. Extracted parameters from consecutive recordings were pooled and analyzed collectively to ensure reliable statistics. For measurements in human serum, traces were cleaned if the voltage was switched and/or if the current drop corresponded to a deep (*∆I_b_* ≥ 0.9) and long (*Dt* ≥ 3 s) current blockade at the same time (Figure S8). For dwell time and frequency analyses, values refer to events selected according to the following criteria: blockade duration, 200 µs to 1 s; blockade amplitude, depending on the biomarker selected; noise, 1 pA to 100 pA. Only events with a duration longer than 200 µs and a blockade level above 0.3 were chosen for statistical analysis. The blockade level histograms were binned at 0.006, and the overall blockade level for different event populations was determined using bi‐Gaussian fits (Figure 2 and Figure S2). The most probable dwell time for events from a single population was determined using a lognormal fit to adjust the number of events versus the event duration (binned at 0.8 ms; Figure S2). To establish the frequency of event population, a semi‐log distribution of interevent time was fit using a single exponential function (Figure S2). At least 1000 events were used to analyze all the data, and the average and standard deviation of individual fits were determined from three independent fits. All experiments presented in this manuscript were realized at least in duplicate.

## Conflicts of Interest

The authors declare no conflicts of interest.

## Supporting information




**Supporting file 1**: smll74968‐sup‐0001‐SuppMat.docx

## Data Availability

The data that support the findings of this study are available from the corresponding author upon reasonable request.
