# Supplementary material for: Identification and Discrimination of Sulfated and Unsulfated Gut‐Hormone Biomarkers in Human Serum by Nanopore
Source: Small. 2026 Aug 3;22(55):e74968. doi: 10.1002/smll.74968 (PMC13630408; doi:10.1002/smll.74968)
Supplement: Supplementary file 1 [file SMLL-22-e74968-s001.docx]

**Supporting Information**

**Identification and Discrimination of Sulfated and Unsulfated Gut-Hormone Biomarkers in Human Serum by Nanopore**

*Luca Iesu^1^, Laurent Bacri^2^, Juan Pelta^2*^, Benjamin Cressiot^1*^*

^1.^ Université Paris-Saclay, Univ. d'Évry, CY Cergy Paris Université, CNRS, LAMBE, 95000 Cergy, France.

^2.^ Université Paris-Saclay, Univ. d'Évry, CY Cergy Paris Université, CNRS, LAMBE, 91025 Évry-Courcouronnes, France.

*corresponding authors.

**Table of Contents**

| Supplementary Figure S1 | 3 |
| --- | --- |
| Supplementary Figure S2 | 4 |
| Supplementary Figure S3 | 5 |
| Supplementary Figure S4 | 6 |
| Supplementary Figure S5 | 7 |
| Supplementary Figure S6 | 8 |
| Supplementary Figure S7 | 9 |
| Supplementary Figure S8 | 10 |
| Supplementary Figure S9 | 11 |
| Supplementary Figure S10 | 12 |
| Supplementary Figure S11 | 13 |
| Supplementary Figure S12 | 14 |
| Supplementary Figure S13 | 15 |
| Supplementary Figure S14 | 16 |
| Supplementary Figure S15 | 17 |
| Supplementary Figure S16 | 18 |
| Supplementary Figure S17 | 19 |

**Supplementary Figure S1:** Independent characterization of diagnostic gut-hormone biomarkers. (a) Normalized average blockade level for unsulfated cholecystokinin-8 (CCK-8) and sulfated cholecystokinin-8 (SO_3_-CCK-8) versus the voltage bias applied, followed by its (b) grey area zoomed. (c) Blockade frequency of CCK-8 (teal) and SO_3_-CCK-8 (magenta), as a function of their concentration, under a fixed +80 mV applied voltage. Dotted lines correspond to the fitted linear function with slopes of (2.07 ± 0.09) × 10^-2^, and (6.63 ± 1.05) × 10^-3^ nM^-1^.s^-1^ for SO_3_-CCK-8 (*R^2^*= 0.99), and CCK-8 (*R^2^*= 0.93), respectively. (d) Blockade frequency of CCK-8 and SO_3_-CCK-8, as a function of the applied voltage. Dotted lines correspond to the fitted linear function with slopes of (9.44 ± 0.35) × 10^-2^, and (2.36 ± 0.25) × 10^-2^ nM^-1^.s^-1^ for SO_3_-CCK-8 (*R^2^*= 0.99), and CCK-8 (*R^2^*= 0.91), respectively. (e) Mean residence time as a function of the voltage applied.

**Supplementary Figure S2:** Characterization of unsulfated cholecystokinin-8 (CCK-8). (a) Typical current trace on a timescale of 5 s. (b) Scatter plot of normalized average blockade level versus the dwell time. Each point corresponds to a distinct blockade event. The dotted rectangle indicates the target population. (c) Histogram of normalized average blockade level versus the normalized events. The black line indicates the bi-Gaussian function used to fit (0.40 ± 0.01). (d) Histogram of the number of events versus event duration; the teal line corresponds to the lognormal function used to fit (8.9 ± 0.14 ms). (e) Count versus inter-event time; the black line corresponds to the fitted exponential function (0.94 ± 0.03 Hz). Fitted values in (d) and (e) correspond exclusively to the population within the selected capture zone, depicted with a blue star.

**Supplementary Figure S3:** Characterization of sulfated cholecystokinin-8 (SO_3_-CCK-8). (a) Typical current trace on a timescale of 5 s. (b) Scatter plot of normalized average blockade level versus the dwell time. Each point corresponds to a distinct blockade event. The dotted rectangle indicates the target population. (c) Histogram of normalized average blockade level versus the normalized events. The black line indicates the bi-Gaussian function used to fit (0.40 ± 0.01). (d) Histogram of the number of events versus event duration; the magenta line corresponds to the lognormal function used to fit (9.36 ± 0.77 ms). (e) Counts versus inter-event time; the black line corresponds to the fitted exponential function (2.12 ± 0.12 Hz). Fitted values in (d) and (e) correspond exclusively to the population within the selected capture zone, depicted with a blue star.

**Supplementary Figure S4:** Characterization of unsulfated cholecystokinin-8 (CCK-8). (a) Typical current trace on a timescale of 5 s. (b) Scatter plot of normalized average blockade level versus the dwell time. Each point corresponds to a distinct blockade event. The dotted rectangle indicates the target population. (c) Histogram of normalized average blockade level versus the normalized events. The black line indicates the Gaussian function used to fit (0.38 ± 0.01). (d) Histogram of the number of events versus event duration; the teal line corresponds to the lognormal function used to fit (67 ± 7 ms). (e) Count versus inter-event time; the black line corresponds to the fitted exponential function (2.92 ± 0.49 Hz). Fitted values in (d) and (e) correspond exclusively to the population within the selected capture zone, depicted with a blue star.

**Supplementary Figure S5:** Characterization of sulfated cholecystokinin-8 (SO_3_-CCK-8). (a) Typical current trace on a timescale of 5 s. (b) Scatter plot of normalized average blockade level versus the dwell time. Each point corresponds to a distinct blockade event. The dotted rectangle indicates the target population. (c) Histogram of normalized average blockade level versus the normalized events. The black line indicates the bi-Gaussian function used to fit (0.41 ± 0.01 and 0.40 ± 0.01). (d) Histogram of the number of events versus event duration; the magenta line corresponds to the lognormal function used to fit (15 ± 0.57 ms). (e) Counts versus inter-event time; the black line corresponds to the fitted exponential function (9.74 ± 0.77 Hz). Fitted values in (d) and (e) correspond exclusively to the population within the selected capture zone, depicted with a blue star.

**Supplementary Figure S6:** Discrimination of CCK-8 (teal) and SO_3_-CCK-8 (magenta) in a mix with different ratios. Varying biomarker concentrations (a), (d), (g) Typical current traces on a timescale of 8 s. (b), (e), (h) Scatter plots of normalized average blockade level versus the dwell time. Each point corresponds to a distinct blockade event. (c), (f), (i) Histograms of normalized average blockade level versus the normalized events. The black line indicates the bi-Gaussian function used for fitting, 0.41 ± 0.01 and 0.38 ± 0.01 for SO_3_-CCK-8 and CCK-8, respectively. Values in (b), (e), and (h) correspond exclusively to the population within the selected capture zone, targeting the desired biomarker population, as done for the independent characterization (S2-S5).

**Supplementary Figure S7:** Discrimination of CCK-8 (teal) and SO_3_-CCK-8 (magenta) in an equimolar mix. Varying voltage condition (a), (d) Typical current traces on a timescale of 8 s. (b), (e) Scatter plots of normalized average blockade level versus the dwell time. Each point corresponds to a distinct blockade event. (c), (f) Histograms of normalized average blockade level versus the normalized events. The black line indicates the bi-Gaussian function used for fitting, 0.41 ± 0.01 and 0.38 ± 0.01 for SO_3_-CCK-8 and CCK-8, respectively. Values in (b) and (e) correspond exclusively to the population within the selected capture zone, targeting the desired biomarker population, as done for the independent characterization (S2-S5).

**Supplementary Figure S8:** Example of full electrical recording (~10 minutes) of wild-type aerolysin reconstituted into a hybrid membrane measuring 20% v/v human serum in 3.2 M KCl, pH 7.5, before the cleaning process. The spikes correspond to manual voltage switching to unclog the aerolysin nanosensor.

**Supplementary Figure S9:** Example of full electrical recording of wild-type aerolysin reconstituted into a hybrid membrane measuring 20% v/v human serum in 3.2 M KCl, pH 7.5, after the cleaning process.

**Supplementary Figure S10:** Continuum of S9.

**Supplementary Figure S11:** Parameter calculation for CCK-8 and SO_3_-CCK-8 biomarkers under different concentrations in human serum conditions. (a), (b), (e), (f) Histogram of the number of events versus the event duration. Teal (47.15 ± 1.35 ms at 250 nM; 49.14 ± 1.35 ms at 62.5 nM) and magenta (80.51 ± 1.43 ms at 250 nM; 89.08 ± 0.98 ms at 62.5 nM) lines correspond to the lognormal functions used for fitting. (c), (d), (g), (h) Histogram of the number of events versus the standard deviation. Teal (11.4 ± 0.44 pA at 250 nM; 11.19 ± 0.62 pA at 62.5 nM) and magenta (6.54 ± 0.19 pA at 250 nM; 5.23 ± 0.13 pA at 62.5 nM) lines correspond to the Gaussian functions used for fitting. Fitted values correspond exclusively to the population within the selected capture zone and target the desired biomarker population, as in the independent characterization (S2-S5).

**Supplementary Figure S12:** Parameter calculation for CCK-8 and SO_3_-CCK-8 biomarkers under different concentrations in buffer conditions. (a), (b), (e), (f) Histogram of the number of events versus the event duration. Teal (16.39 ± 1.14 ms at 250 nM; 15.67 ± 1.14 ms at 125 nM) and magenta (38.99 ± 1.69 ms at 250 nM; 35.93 ± 2.44 ms at 125 nM) lines correspond to the lognormal functions used for fitting. (c), (d), (g), (h) Histogram of the number of events versus the standard deviation. Teal (11.36 ± 0.36 pA at 250 nM; 9.76 ± 0.47 pA at 125 nM) and magenta (5.37 ± 0.12 pA at 250 nM; 4.02 ± 0.21 pA at 125 nM) lines correspond to the Gaussian functions used for fitting. Fitted values correspond exclusively to the population within the selected capture zone and target the desired biomarker population, as in the independent characterization (S2-S5).

**Supplementary Figure S13:** Parameter calculation for CCK-8 and SO_3_-CCK-8 biomarkers under different concentrations in buffer conditions. (a), (b), (e), (f) Histogram of the number of events versus the event duration. Teal (15.48 ± 1.32 ms at 62.5 nM; 19.65 ± 1 ms at 30 nM) and magenta (39.81 ± 2.13 ms at 62.5 nM; 35.33 ± 1.7 ms at 30 nM) lines correspond to the lognormal functions used for fitting. (c), (d), (g), (h) Histogram of the number of events versus the standard deviation. Teal (9.14 ± 0.52 pA at 62.5 nM; 8.89 ± 0.43 pA at 30 nM) and magenta (4.46 ± 0.1 pA at 62.5 nM; 4.54 ± 0.13 pA at 30 nM) lines correspond to the Gaussian functions used for fitting. Fitted values correspond exclusively to the population within the selected capture zone and target the desired biomarker population, as in the independent characterization (S2-S5).

**Supplementary Figure S14:** Parameter calculation for CCK-8 and SO_3_-CCK-8 biomarkers under different concentrations in serum conditions. (a), (e) Typical current traces on a 15 s timescale. (b), (f) Histograms of normalized average blockade level versus the normalized events. Black lines correspond to the bi-Gaussian function used for fitting, 0.41 ± 0.01 and 0.38 ± 0.01 for SO_3_-CCK-8 and CCK-8, respectively. (c), (d), (g), (h) Number of events versus the inter-event time. Black lines correspond to the exponential functions used for fitting. At 250 nM: 0.59 ± 0.01 Hz and 2.06 ± 0.07 Hz, for CCK-8 and SO_3_-CCK-8, respectively. At 62.5 nM: 0.11 ± 0.01 Hz and 0.32 ± 0.02 Hz, for CCK-8 and SO_3_-CCK-8, respectively. Fitted values correspond exclusively to the population within the selected capture zone and target the desired biomarker population, as in the independent characterization (S2-S5).

**Supplementary Figure S15:** Parameter calculation for CCK-8 and SO_3_-CCK-8 biomarkers under different concentrations in buffer conditions. (a), (e) Typical current traces on a 15 s timescale. (b), (f) Histograms of normalized average blockade level versus the normalized events. Black lines correspond to the bi-Gaussian function used for fitting, 0.41 ± 0.01 and 0.38 ± 0.01 for SO_3_-CCK-8 and CCK-8, respectively. (c), (d), (g), (h) Number of events versus the inter-event time. Black lines correspond to the exponential functions used for fitting. At 250 nM: 0.58 ± 0.03 Hz and 2.47 ± 0.23 Hz, for CCK-8 and SO_3_-CCK-8, respectively. At 125 nM: 0.42 ± 1 Hz and 1 ± 0.06 Hz, for CCK-8 and SO_3_-CCK-8, respectively. Fitted values correspond exclusively to the population within the selected capture zone and target the desired biomarker population, as in the independent characterization (S2-S5).

**Supplementary Figure S16:** Parameter calculation for CCK-8 and SO_3_-CCK-8 biomarkers under different concentrations in buffer conditions. (a), (e) Typical current traces on a 15 s timescale. (b), (f) Histograms of normalized average blockade level versus the normalized events. Black lines correspond to the bi-Gaussian function used for fitting, 0.41 ± 0.01 and 0.38 ± 0.01 for SO_3_-CCK-8 and CCK-8, respectively. (c), (d), (g), (h) Number of events versus the inter-event time. Black lines correspond to the exponential functions used for fitting. At 62.5 nM: 0.15 ± 0.01 Hz and 0.67 ± 0.02 Hz, for CCK-8 and SO_3_-CCK-8, respectively. At 30 nM: 0.11 ± 0.01 Hz and 0.41 ± 0.01 Hz, for CCK-8 and SO_3_-CCK-8, respectively. Fitted values correspond exclusively to the population within the selected capture zone and target the desired biomarker population, as in the independent characterization (S2-S5).

**Supplementary Figure S17:** Gut-hormone biomarker discrimination in human serum conditions at two different concentrations. Mean residence time as a function of the standard deviation; CCK-8, in teal, SO_3_-CCK-8, in magenta.
